# Traction-bed-assisted reduction and double-plate fixation for treatment of comminuted femoral intertrochanteric fractures with coronal split

**DOI:** 10.3389/fsurg.2022.984431

**Published:** 2022-09-09

**Authors:** Liangcong Hu, Xudong Xie, Tiantian Wang, Bobin Mi, Hang Xue, Ze Lin, Yuan Xiong, Yiqiang Hu, Wu Zhou, Faqi Cao, Guohui Liu

**Affiliations:** ^1^ Department of Orthopedics, Union Hospital, Tongji Medical College, Huazhong University of Science and Technology, Wuhan, China; ^2^ Department of Emergency, Union Hospital, Tongji Medical College, Huazhong University of Science and Technology, Wuhan, China

**Keywords:** internal fixation, femoral, intertrochanteric fracture, coronal fracture, nonunion

## Abstract

**Background:**

A coronal comminuted femoral intertrochanteric fracture is a special type of fracture that easily leads to internal fixation failure, and the current internal fixation techniques remain controversial. This study aims to evaluate the effect of traction-bed-assisted reduction and double-plate internal fixation in the treatment of comminuted and coronally split intertrochanteric femoral fracture.

**Method:**

Retrospective analyses of the clinical data of 83 patients diagnosed with, and treated for, comminuted and coronally split intertrochanteric femoral fracture from December 2017 to November 2019 were conducted. Among the total number of 83 patients, 40 patients received traction-bed-assisted reduction and PFNA fixation (the control group), whereas 43 patients received traction-bed-assisted reduction and double-plate internal fixation (the experimental group). The major indicators for the research analysis such as the general information of patients, perioperative data, and follow-up data of both groups were collected, sorted out, and meticulously analyzed.

**Results:**

The time taken for traction-bed-assisted reduction and double-plate intern fixation in the experimental group was significantly shorter than that in the control group (*P* < .05). The post-operative Harris Hip Score (HHS) at 3 months and at the final follow-up after the surgery was significantly better in the experimental group compared with that in the control group, both of which were statistically significant (*P* < .05). However, there were statistically no significant differences between the two groups in terms of preoperative hemoglobin (Hb) level, amount of intraoperative total blood loss, immediate post-operative Hb level, incidence of wound infection within 14 days post-operatively, time taken to step up on the ground after surgery, HHS 2 weeks after surgery, time taken for fracture healing, and the incidence of complications (*P* > .05).

**Conclusion:**

The use of a traction bed to achieve adequate reduction, followed by internal fixation using double plates, comparatively takes less time for both reduction and operation in the treatment of comminuted and coronally split intertrochanteric femoral fractures, which also restores proper hip joint movements relatively early and hence provides better hip joint functions in the long run.

## Introduction

It is estimated that the global incidence of hip fractures will increase from 126,000 in 1990 to 450,000 by 2050. The average age of hip fractures is 80 ± 11 years, and women account for 69% and AO 31-A2 type accounts for up to 49% (1, 2). Most patients with hip fractures are elderly people, with underlying diseases such as osteoporosis and diabetes. Early intervention enables patients to perform early functional exercises, which could significantly reduce complications caused by prolonged bed rest and improve the quality of life of patients as soon as possible. Recent studies have pointed out that the mortality rate of conservative treatment of hip fracture for 3 months is as high as 30% (3–6). The fracture healing rate of conservative treatment is 23.8%, which is lower than that of surgical treatment, and the incidence of ischemic necrosis is 3.4% higher, which easily leads to a transition from simple fractures to complex fractures and increases the incidence of fracture malunion (7, 8). Compared with non-surgical treatment, surgical treatment significantly increases the healing rate. However, iatrogenic injury, fracture nonunion rate, and infection caused by surgical treatment remain high due to the failure of internal fixation (9, 10). Therefore, it is necessary to explore better fixation methods to prevent the occurrence of these disadvantages.

The current fixation way for intertrochanteric fractures combined with coronal fractures mainly include: (1) a gradient ring steel plate, (2) intramedullary nail combined with wire cerclage, and (3) lag screw fixation and use of the greater trochanter fixator to treat the inner coronal surface fracture block (11–13), but none of the above methods can guarantee sufficient mechanical stability and bridging extrusion. Combining the characteristics of the posterior and lateral anatomical structure of the greater trochanter and the mechanical stability requirements for fracture fixation, we used double plates to treat intertrochanteric fractures with coronal splitting. Clinical observations have found that it could achieve good reduction and fixation maintenance effects with no increased operation time, blood loss, and other complications.

## Materials and methods

### Inclusion criteria

(a) Closed intertrochanteric femoral fractures of up to 1 week, (b) Intertrochanteric femoral fractures of classification AO 31-A2.2 to A2.3, (c) Patients aged over 50 years old, (d) Patients with undamaged other organs, (e) Intertrochanteric femoral fractures treated with either PFNA or double-plate internal fixation.

### Exclusion criteria

(a) Intertrochanteric femoral fractures older than 1 week, (b) Open intertrochanteric femoral fractures, (c) Patients having functional limitations of the hip joint prior to the intertrochanteric femoral fracture, (d) Intertrochanteric femoral fractures treated with single-plate internal fixation, (e) Intertrochanteric femoral fractures associated with serious underlying diseases contraindicating surgical treatment, (f) Patients diagnosed with intertrochanteric femoral fracture but who voluntarily refused surgery.

The Committee of Clinical Ethics of the Union Hospital, Tongji Medical College, Huazhong University of Science and Technology (HUST), Wuhan, China, approved all the research studies.

#### Basic information

In accordance with the inclusion and exclusion criteria, we retrospectively analyzed the clinical data of 83 patients diagnosed with, and treated for, comminuted and coronally split intertrochanteric femoral fracture from December 2017 to November 2019 in the Department of Orthopedics, Union Hospital, Tongji Medical College, Huazhong University of Science and Technology (HUST), Wuhan, China. Out of the total number of 83 patients, 40 received traction-bed-assisted reduction and PFNA fixation (the control group), whereas 43 received traction-bed-assisted reduction and double-plate internal fixation (the experimental group). Statistical parameters of general information of the two groups of patients included their age, gender, total body weight, and length of follow-up time.

#### Observation indicators

### Perioperative indicators

(a) *Preoperative:* AO classification of intertrochanteric femoral fracture of the hip joint, hemoglobin (Hb) level, erythrocyte sedimentation rate (ESR), C-reactive protein (CRP), and white blood cell (WBC) count. (b) *Intraoperative:* time taken for traction-bed-assisted reduction (reduction time), time taken for surgical operation of internal fixation (operating time), and the amount of total blood loss. (c) *Post-operative:* Immediate post-operative Hb level, ESR at 1 (one) week after surgery, CRP at 1 (one) week after surgery, WBC count at 1 (one) week after surgery, incidence of wound infection within 14 days post-operatively, time taken to step up on the ground (ground-stepping time) after surgery, and lateral view radiograph (x-ray) of the hip joint at 2 weeks after surgery to assess the quality of reduction and internal fixation.

### Follow-up indicators

(a) Clinical assessment at 2 weeks and at 3 months after surgery, (b) Harris Hip Score (HHS) of the hip joint at the final follow-up after the surgery in the later stage of treatment, (c) time taken for fracture healing, and (d) any complication of the internal fixation till the final follow-up after surgery.

#### Surgical methods

### The control group

After administering adequate general anesthesia to the patient, a traction bed was used to achieve the desired amount of reduction of the intertrochanteric femoral fracture, which was verified instantly through a series of real-time static and motional images taken by portable bedside x-ray fluoroscopy, and upon satisfactory fluoroscopic verification of reduction of the fracture, the traction-bed-assisted force of traction to maintain the acquired reduction was set in persistent continuity. By keeping the patient engaged on the traction bed clamped to the operating table, essential antiseptic paints followed by sterile draping were applied, focusing around the site of the planned surgical intervention.

Approximately 6–8 cm of longitudinal incision on the upper lateral side of the hip joint was made. The skin, subcutaneous tissues, and the layers beneath were dissected layer after layer. The gluteus maximus muscle was carefully split apart to expose the proximal apex of the greater trochanter of the femur bone. A preselected radio-opaque guide wire was planted in the starting position on the tip of the greater trochanter of the femur as ascertained by the exact calculation of the caput-collum-diaphyseal (CCD) angle using apposite accessories. After visualizing the desired predictive direction of insertion of the guide wire under static and motional fluoroscopic images taken on the spot, the guide wire was driven deep into the greater trochanter of the femur up to the femoral shaft by using a power tool, drill sleeve, protection sleeve, and other accessories. With continuous watch under x-ray fluoroscopy, a centrally cannulated reaming rod was driven over the guide wire, and the internal medullary canal of the proximal femur was reamed out up to the required length. Then, after a satisfactory fluoroscopic verification of the result of reaming activity, the reaming rod was withdrawn. Now, the most suitable tubular proximal femoral intramedullary nail was selected and driven over the guide wire deep into the greater trochanter of the femur up to the femoral shaft by using an insertion handle and other essential tools of the set with continuous monitoring under x-ray fluoroscopy. Upon a satisfactory fluoroscopic verification of the fixation of the tubular proximal femoral intramedullary nail, the guide wire was eventually removed. The angle formed by the axes of the femoral neck and femoral shaft was adjusted to the proper anatomical position *via* a fluoroscopic image-navigated surgical maneuver. Real-time static and motional fluoroscopic images were repeatedly taken whenever required throughout the procedure.

Another longitudinal incision of approximately 4–5 cm was made on the upper lateral side of the thigh. After dissecting through the layers, the required portion of the femur was exposed and identified, which allowed access to the neck of the femur and eventually to the head of the femur. Next, a preselected radio-opaque guide wire was stabbed a tad in the starting position on the exact site identified over the lateral proximal femur for driving it obliquely into the neck of the femur after being ascertained by the exact calculation of caput-collum-diaphyseal CCD angle. After visualizing the favorable predictive path of insertion of the guide wire through a series of real-time fluoroscopic shots taken on the spot, the guide wire was inserted obliquely deep into the neck of the femur and ultimately leading up to the head of the femur under fluoroscopic guidance by using an insertion handle, aiming arm, power tool, drill sleeve, and other accessories. A centrally cannulated reamer was driven over the guide wire into the femoral neck and femoral head, up to the required length, and soon after the satisfactory fluoroscopic verification of the result of reaming activity, the reamer was withdrawn. Assured of the right direction as confirmed through a series of real-time static and motional fluoroscopic images, a ready-made centrally cannulated self-tapping proximal locking lag screw was selected and driven obliquely over the guide wire deep into the neck of the femur leading up to the head of the femur, ultimately compressing the intertrochanteric femoral fracture, as well as crossing and interlocking with the vertical proximal femoral intramedullary nail that was inserted through the greater trochanter of the femur just earlier. After a satisfactory fluoroscopic verification of the fixation of the proximal locking lag screw, the guide wire was eventually removed. Last but not least, a single ready-made static distal locking screw was selected and driven more or less perpendicular to the femoral shaft, crossing and interlocking with the distal part of the proximal femoral intramedullary nail placed just earlier inside the greater trochanter and mid-shaft of the femur, thereby preventing any possible rotation. The proximal end cap was placed atop the proximal femoral intramedullary nail and its hollow interior was shut up.

The entire procedure of Proximal Femoral Nail Antirotation (PFNA) fixation was monitored, rectified, and verified under several real-time static and motional fluoroscopic images repeatedly taken on the spot throughout the procedure until the final successful results were achieved. Before wrapping up the surgery, all the opened sites of operation were flushed with normal saline and cleaned thoroughly. Any overlooked bleeding sites were tackled and satisfactory hemostasis was secured. A temporary negative-pressure surgical drain was placed wherever deemed necessary. All the operated sites were double-checked before the surgical wounds were closed in layers using proper suturing materials and techniques. Finally, the wounds were dressed up, and the patient was reinstated to consciousness.

### The experimental group

After administering adequate general anesthesia to the patient, a traction bed was used to achieve the desired amount of reduction of the intertrochanteric femoral fracture, which was verified instantly through a series of real-time static and motional images taken by portable bedside x-ray fluoroscopy. Upon a satisfactory fluoroscopic verification of the reduction of the fracture, the traction-bed-assisted force of traction to maintain the acquired reduction was set in persistent continuity. By keeping the patient engaged on the traction bed clamped to the operating table, essential antiseptic paints, followed by sterile draping, was applied, focusing around the site of the planned surgical intervention.

A longitudinal incision of approximately 10 cm was made on the skin lying precisely above along the proximal femur and the greater trochanter of the femur, so as to expose it anterio-laterally. The underlying structures of subcutaneous tissues, fascia lata, and so on were dissected layer after layer, while the fibers of the lateral femoral muscle were split apart carefully. The internally injured site was explored, the femoral fracture was identified, and the fractured site was sufficiently exposed for taking action. The hematoma surrounding the fractured site was cleaned up, and the adjacent soft tissues were gently compressed. The intertrochanteric femoral fracture was manually reduced and reset to its most favorable natural position under the direct vision of the naked eye. A titanium plate with 4–5 holes was placed over the neck of the femur anterio-medially spreading up to the greater trochanter of the femur so as to cover the fractured block of bones coronally split apart and was fixed in place with lag screws that strengthened the proximal femur medially. The successful fixation of the plate was verified through a series of real-time static and motional fluoroscopic images taken before, during, and after the attempt on the spot instantly. The next titanium-made proximal femur anatomical locking compression plate was placed over much of the proximal part of the femur laterally and was fixed in place with screws. The successful fixation of the second plate was also verified through a series of real-time static and motional fluoroscopic images taken before, during, and after the attempt on the spot instantly.

Before wrapping up the surgery, all the opened sites of operation were flushed with normal saline and cleaned thoroughly. Any overlooked bleeding sites were tackled and satisfactory hemostasis was secured. A temporary negative-pressure surgical drain was placed wherever deemed necessary. All the operated sites were double-checked before the surgical wounds were closed in layers using proper suturing materials and techniques. Finally, the wounds were dressed up, and the patient was reinstated to consciousness.

#### Perioperative management

Routine blood tests, ESR, and CRP were rechecked at 24 h after the surgery. An intravenous administration of Dezocine injection (0.8 mg/kg body weight, q12h) was started at 48 h after the surgery, and later, it was substituted by oral Indomethacin 25 mg tablet (q8h, and PRN) for a few days to control and provide relief from post-operative pain. Flucloxacillin Sodium 500 mg capsule per orally (PO) twice daily/bis in die (BID) was instructed at 72 h after the surgery for a week or two to prevent any susceptible infection. Drugs to protect the stomach and duodenum from any potentially harmful substances as a result of substantial perioperative fasting were administered intravenously and/or orally as required. Likewise, utmost medical care was taken to prevent any thrombosis or bedsores as a consequence of the prolonged bed-ridden position. The surgical wounds were carefully inspected, cleaned, and dressed up anew every 2 days after the surgery until all the stitches on the incised skin were removed 2 weeks after the surgery. Optional negative-pressure surgical drains, if any, were uprooted tenderly approximately 48 h after the cessation of any significant collection therein.

#### Post-operative rehabilitation and physical therapy

The patient was instructed to contract and relax the calf muscle of the operated leg independently 12 h after the surgery. The foot of the operated leg was fastened to a “T” shoe 24 h after the surgery. Similarly, the patient was instructed to perform mild flexion and extension of the knee joint and some hip muscle-strengthening exercise by mobilizing the operated limb 24 h after the surgery. The patient was then asked to raise the operated leg up straight without flexing the corresponding knee joint 72 h after the surgery. The patient was allowed to bear weight equivalent to 1/8th of the weight of the operated limb by stepping up on the ground with some assistance 3 days after the surgery. Furthermore, the patient was encouraged to bear a little more weight equivalent to 1/6th of the weight of the operated limb by stepping up on the ground without any assistance or support 1 week after the surgery. The patient was advised to perform gentle adduction and abduction exercise by mobilizing the operated leg independently after being discharged from the hospital. The amount of prescribed weight and the length of weight-bearing time were calculated and determined according to the actual conditions based on medical reviews.

#### Statistical analysis

The data are expressed as mean ± SD. Statistical analyses were performed by using GraphPad® Prism® 7.0 software (developed by GraphPad Software Inc., CA, USA). The differences between the means of the groups were analyzed by using the one-way Student's *t*-test and one-way ANOVA. The symbols and numericals are as follows: *P* < .05 indicates statistically “significant”, whereas *P < .05, **P < .01, ***P < .001, and NS indicate statistically “not significant”.

## Results

### General information

In comparison, the general information of patients from the two groups, which included their age, gender, total body weight, and the length of follow-up time, was statistically not significant (*P* > .05); see Table 1.

**Table 1:** General conditions and preoperative items.

Category							
Group	Control group			Experimental group			*P*-value
	Mean	SD	Number	Mean	SD	Number	
Age (years)	64.3	6.78	40	63.23	7.15	43	0.48
Gender	–	Male/Total 51.22%	40	–	Male/Total 47.73%	43	0.66
Body weight (kg)	62.1	7.99	40	60.2	7.77	43	0.28
Follow-up time (months)	9.37	1.95	40	9.52	1.64	43	0.73

### Perioperative data

#### Preoperative indicators

The differences in AO Classification of fracture, Hb level, preoperative ESR, preoperative CRP test value, and preoperative WBC count among the two groups of patients were statistically not significant (*P* > .05); see Table 2.

**Table 2:** Perioperative items.

Category								
Group	Control group			Experimental group			*T*-value	*P*-value
	Mean	SD	Number	Mean	SD	Number		
AO classification		2.2 type/total 45.24%	40		2.2 type/total 48.72%	43		0.99
Preoperative Hb	115.15	1.87	40	113.44	2.03	43	0.61	0.54
Preoperative ESR	30.25	7.48	40	30.30	7.691	43	0.03	0.97
Preoperative CRP	1.04	0.61	40	0.96	0.78	43	0.46	0.64
Preoperative WBC	6.91	1.62	40	6.3	1.55	43	1.71	.08
Operative time (min)	91.77	1.31	40	104.6	3.08	43	3.92	.0002
Reduction time (min)	40.23	1.93	40	50.23	2.21	43	3.38	.0011
Intraoperative blood loss (ml)	252.4	27.39	40	265.27	35.44	43	1.84	.06
Post-operative first Hb	96.92	12.97	40	97.34	9.11	43	0.17	0.86
WBC 1 week after surgery	7.20	1.66	40	6.97	2.09	43	0.53	0.59
Post-operative 1 week ESR	40.5	8.91	40	38.93	6.44	43	0.92	0.35
Post-operative 1 week CRP	2.3675	1.53	40	3.01	2.01	43	1.62	0.10
Land time after surgery (days)	6.975	2.32	40	6.74	1.89	43	0.49	0.62

Hb, hemoglobin; ESR, erythrocyte sedimentation rate; CRP, C-reactive protein; WBC, white blood cell.

#### Intraoperative indicators

The length of time taken for traction-bed-assisted reduction (reduction time) and the length of time taken for the surgical operation of internal fixation (operating time) in the experimental group were shorter than those of the control group, which was statistically significant (*P* < .05). However, the difference between the two groups in terms of the total amount of blood loss intraoperatively was statistically not significant (*P* > .05); see Table 2.

#### Post-operative indicators

The differences between the two groups in terms of immediate post-operative Hb level, ESR at 1 week post-operatively, CRP test value at 1 week post-operatively, WBC count at 1 week post-operatively, incidence of wound infection within 2 weeks post-operatively, and the time taken to step up on the ground (ground-stepping time) for the first time after the surgery were statistically not significant (*P* > .05); see Table 2.

**Figure 1:**
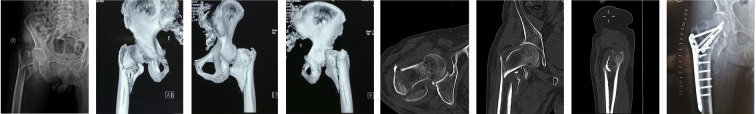
Treatment of intertrochanteric fractures with a split greater trochanter using double plates.

**Figure 2:**
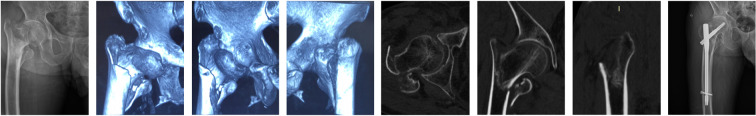
Treatment of intertrochanteric fractures with a split greater trochanter using Proximal Femoral Nail Antirotation (PFNA).

Typical images pertinent to the experimental group are shown in Figure 1, and those pertinent to the control group are shown in Figure 2.

### Follow-up data

#### Follow-up indicators

**Figure 3:**
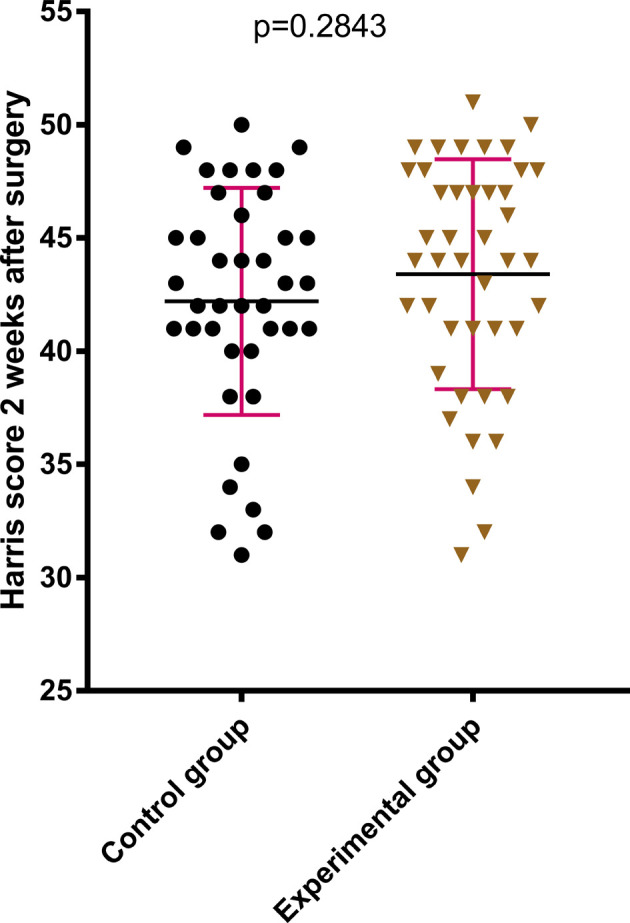
There was no significant difference between the two groups in terms of the Harris score of the hip at 2 weeks after surgery.

**Figure 4:**
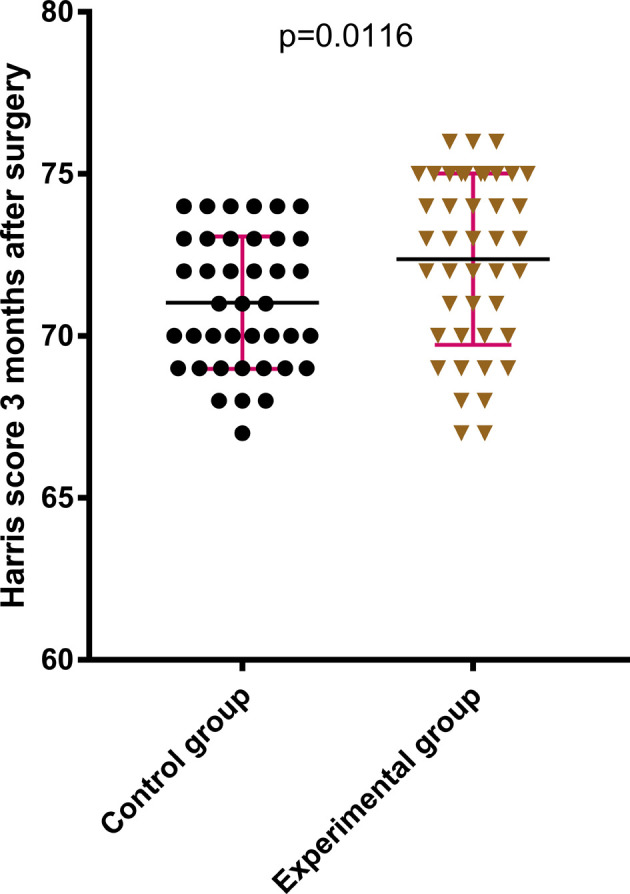
The Harris score of the hip joint at 3 months after operation in the experimental group is better than that in the control group.

**Figure 5:**
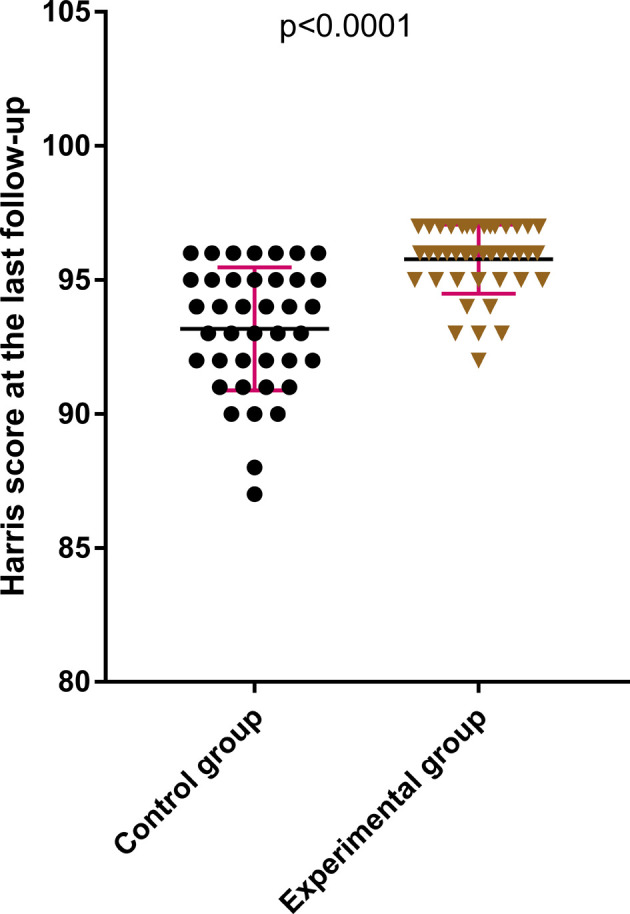
The last Harris score of the hip joint in the experimental group is better than that in the control group.

**Figure 6:**
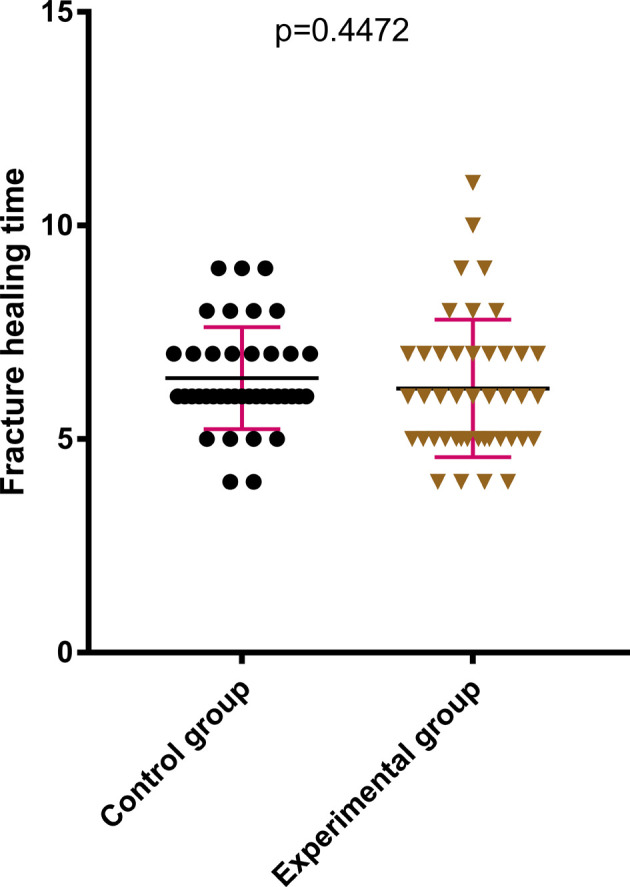
There was no significant difference in fracture healing time between the two groups.

The difference between the two groups in terms of HHS of the hip joint 2 weeks after the surgery was statistically not significant (*P* > .05); see Figure 3. The HHS of the hip joint 3 months after the surgery and the final follow-up after the surgery in the later stage of treatment were much better in the experimental group compared with those in the control group, which were statistically significant (*P* < .05); see Figures 4, 5. The difference in time taken for fracture healing between the two groups was statistically not significant (*P* > .05); see Figure 6.

Unfortunately, during the final follow-up period, three patients of the control group treated with PFNA fixation encountered some least expected traumatic events. The first of these patients sustained a re-fracture of the distal femur, which was operated again, and the existing nail was replaced with a long femoral intramedullary nail. The second one endured a reverse intertrochanteric femoral fracture, which was also treated with a second surgery by replacing the existing nail with a long femoral intramedullary nail, both of which later healed smoothly. The third one suffered a femoral head fracture, which was successfully treated through revision surgery by retracting the existing PFNA fixation, followed by double-plate internal fixation that included the “proximal femoral anatomical locking compression plate” as one of its indispensable components.

Among those treated with double-plate internal fixation in the experimental group, one case of rare deformity with regard to the shortening of the femoral neck was dealt successfully without any necessity for special intervention. None of the patients from the experimental group succumbed to any major post-operative complications such as infection or re-fracture.

## Discussion

The anterior side of the fracture line passes through the intertrochanteric spine, and the distal coronal fracture line passes within 1 cm below the lesser trochanter. The split with the greater trochanter and the femoral neck is an intertrochanteric fracture with coronal split (14). According to the AO classification, it belongs to type A 31-A2.2 to A2.3; in the Evans–Jensen classification, it belongs to type 3 or type 4, which is characterized by a poor force line and unstable fracture after reduction, and both belong to the category of unstable fractures (15–17). The coronal fracture of the femoral trochanter is easily missed by x-ray examination. The three-dimensional reconstruction of CT scan can significantly improve diagnostic accuracy and reduction success rate (14, 18). The integrity of the inner and outer walls of the femur plays an important role in maintaining the stability of the reduction. Due to the special local anatomy, the iliopsoas muscle, quadratus femoris, and obturator external muscle traction on the posterior coronal bone mass, the coronal micro passer is prone to anterior and proximal displacements, resulting in loss of posterior medial support. When the fracture line of the distal end of the trabecular surface exceeds the small passer and combines with femoral shaft fractures, the support ability and anti-rotation ability of the inner wall of the femur will decrease. The thickness of the outer wall of some elderly patients is less than 20.5 mm, and internal fixation should not be treated with DHS alone (19–22). The blood supply of the base of the femur is mainly supplied by the medial and lateral femoral circumflex artery, the nourishing blood vessels inside the femur, and the blood supply from the femoral neck (23). When the femoral trochanter is combined with a large coronal fracture on the posterior side of the greater trochanter, it may affect the blood of the medial femoral circumflex artery and the blood flow inside the femur, thereby affecting the healing of fractures.

The main factors influencing the internal fixation method include local bone quality, fracture type, and required mechanical strength of fixation (24, 25). For intertrochanteric fractures, both intramedullary fixation and extramedullary fixation can achieve good fixation effects, but for the special type of AO 31-A2, and because the incidence of fixation failure is relatively high, there are still many options for internal fixation of the dispute (26). The biggest controversy is how to restore the support strength and mechanical stability of the inner and outer side walls. Bin Yu et al. (27) reported that it is possible to increase the mechanical strength and stability by placing lag screws deeper and closer to the cortex by specifically improving the accuracy of the fixation of the internal fixation, but too long intramedullary nails also increase the possibility of stress fracture of the femoral shaft (28). In addition, some studies believe that reduction and extramedullary fixation under direct vision can significantly increase the quality and effect of reduction compared with intramedullary fixation (29). It has been reported that double-plate fixation is mostly used for the treatment of periprosthetic fractures of the distal femur, periprosthetic fractures of the proximal femur, and intertrochanteric fractures and is a remedy for failure of intramedullary nail fixation (30, 31). But we are the first to use this technique in intertrochanteric fractures with coronal split.

Based on the principles of anatomical reduction and reconstruction of mechanical stability, we performed the most appropriate anatomical reduction of the greater trochanteric fracture and its posterior block of fractured bones coronally split apart from the femoral neck. Through the lateral longitudinal incision on the area over the greater trochanter of the proximal femur, the comminuted and coronally split intertrochanteric femoral fracture was approached, manually reduced, and internally fixed with double (two) plates. The most suitable proximal femur anatomical locking compression plate was placed and fixed just about vertically (or longitudinally) on the proximal part of the femur laterally, thereby compressing and reinforcing the intertrochanteric femoral fracture. Another most suitable titanium-made locking compression plate was placed and fixed anterio-medially, more or less in an oblique manner on the femoral neck spreading over the posterior fractured block of trochanteric bones coronally split apart from the femoral neck and thus compressing them altogether. This kind of double-plate internal fixation compresses the intertrochanteric femoral fractures adequately, maintains impeccable mechanical stability, and restores the hip joint functions comparatively early. Although we have demonstrated the success of the double-plate internal fixation for the comminuted and coronally split intertrochanteric femoral fractures in a limited number of clinical cases, the follow-up time was not long; finally, there was a lack of *in vitro* mechanical data measurement support. Further large-scale clinical trials and long-term clinical observations would be worthwhile to screen out discernible bias and expose other unknowns.

## Conclusion

Comminuted and coronally split intertrochanteric femoral fractures have a high rate of incidence, and unfortunately, there are equally high chances of such fractures being missed during the diagnosis given to the elusive anatomical position of the posterior trochanter split apart from the femoral neck along the coronal plane, which is hardly noticeable in conventional 2D radiographs or x-rays, unless a dynamic 3D CT or an equivalent image of the fracture is created and analyzed from all angles. The supporting force of the greater trochanter and posterior wall of the proximal femur against such internal fixations, for instance, simple intramedullary nailing or single locking compression plating, is highly compromised, thus resulting in its failure. But contrastingly, traction-bed-assisted reduction, followed by the “double-plate” internal fixation, compresses the intertrochanteric femoral fractures adequately, maintains the supporting force of the greater trochanter and posterior wall of the proximal femur significantly, and most importantly, compensates for any mechanical instability. Therefore, it can restore proper hip joint movements relatively early and provide better hip joint functions in the long run. This is indeed an innovative method of internal fixation worth promoting.

## Data Availability

The original contributions presented in the study are included in the article/Supplementary Material, and further inquiries can be directed to the corresponding author/s.
